# Mechanical, Thermal, Electrical Characteristics and EMI Absorption Shielding Effectiveness of Rubber Composites Based on Ferrite and Carbon Fillers

**DOI:** 10.3390/polym13172937

**Published:** 2021-08-31

**Authors:** Ján Kruželák, Andrea Kvasničáková, Klaudia Hložeková, Roderik Plavec, Rastislav Dosoudil, Marek Gořalík, Jarmila Vilčáková, Ivan Hudec

**Affiliations:** 1 Department of Plastics, Rubber and Fibres, Faculty of Chemical and Food Technology, Slovak University of Technology in Bratislava, Radlinského 9, 812 37 Bratislava, Slovakia; andrea.kvasnicakova@stuba.sk (A.K.); klaudia.hlozekova@stuba.sk (K.H.); roderik.plavec@stuba.sk (R.P.); ivan.hudec@stuba.sk (I.H.); 2 Department of Electromagnetic Theory, Faculty of Electrical Engineering and Information Technology, Slovak University of Technology in Bratislava, Iľkovičova 3, 812 19 Bratislava, Slovakia; rastislav.dosoudil@stuba.sk; 3 Polymer Centre, Faculty of Technology, Tomas Bata University in Zlín, Vavrečkova 275, 760 01 Zlín, Czech Republic; goralik@utb.cz; 4 Centre of Polymer Systems, University Institute, Tomas Bata University in Zlín, Třída Tomáše Bati 5678, 760 01 Zlín, Czech Republic; vilcakova@utb.cz

**Keywords:** absorption shielding, electromagnetic interference, manganese-zinc ferrite, carbon nanotubes, carbon black

## Abstract

In this work, rubber composites were fabricated by incorporation of manganese-zinc ferrite alone and in combination with carbon-based fillers into acrylonitrile-butadiene rubber. Electromagnetic parameters and electromagnetic interference (EMI) absorption shielding effectiveness of composite materials were examined in the frequency range 1 MHz–3 GHz. The influence of ferrite and fillers combination on thermal characteristics and mechanical properties of composites was investigated as well. The results revealed that ferrite imparts absorption shielding efficiency to the composites in tested frequency range. The absorption shielding effectiveness and absorption maxima of ferrite filled composites shifted to lower frequencies with increasing content of magnetic filler. The combination of carbon black and ferrite also resulted in the fabrication of efficient EMI shields. However, the EMI absorption shielding effectiveness was lower, which can be ascribed to higher electrical conductivity and higher permittivity of those materials. The highest conductivity and permittivity of composites filled with combination of carbon nanotubes and ferrite was responsible for the lowest absorption shielding effectiveness within the examined frequency range. The results also demonstrated that combination of ferrite with carbon-based fillers resulted in the enhancement of thermal conductivity and improvement of mechanical properties.

## 1. Introduction

Electromagnetic interference can be defined as a kind of environmental pollution produced by electronic, radiation and electro-communication devices. The rapid progress in modern technology is connected with higher accumulation of EMI in the surrounding. It has been revealed that undesired EMI can lead to the lowering of efficiency of electronic equipment, even to their malfunctions [1,2]. Not least, negative impact of EMI on human beings’ health and ambient has been reported as well [3,4]. Therefore, efficient methods and materials have been developed to reduce EMI in the surrounding and thus to protect the functionality of electronic devices and human health. 

In general, the combination of three shielding mechanisms plays the crucial role in reducing or eliminating of EMI. Those mechanisms are absorption, reflection and multiply reflection of electromagnetic radiation [5,6,7]. Reflection is basic shielding mechanism that relates to a simple reflection of EMI from the outer surface of the shield. To shield EMI by reflection, the material must have free electric charges (electrons or holes), which interfere with electromagnetic plane wave in the radiation [8]. On the other hand, necessary condition of absorption shielding is the presence of electric or magnetic dipoles in the shield. Multiply reflection takes part as the third shielding mechanism and refers to reflection of EMI from internal surfaces, interfaces and various inhomogeneities in the shielding material [9,10]. Foam and porous materials exhibit high specific surface, while large phase interfaces have composites filled with different types of fillers. Such materials usually show good ability of multiply reflection. 

Formerly, metal-based plates were extensively used as efficient EMI shields due to their unique electrical conductivity. However, heavy processing and manipulation, low flexibility and heaviness limit the utilization of metal-based shields. In addition, possessing high electrical conductivity, the reflection of EMI is the most common shielding mechanism for metal-based shields [11,12]. The reflected electromagnetic waves can interfere with primary radiation that is emitted from neighboring electronic appliances, causing the so called collateral electromagnetic radiation effect. Composites based on polymer matrices and suitable filler inclusions can be used as alternatives to traditional metal-based EMI shields due to their low weight, high flexibility and elasticity, good process-ability, corrosion resistance, tunable properties or low cost. They can be shaped, bent, coiled without the loss of their electromagnetic properties. Moreover, due to appropriate electrical and dielectric characteristics, they are able to avert electrostatic discharge, disturbance and interference between the electronic systems. Having the outlined advantages, polymer composites have been used in practical applications in the sphere of automobiles, aircraft, aerospace, civil engineering or commonly used electronic devices [13]. The adjusting of electromagnetic characteristics to polymer matrices can be performed by selection of suitable fillers. The application of fillers into polymer matrices enables not only to tune mechanical and structural properties of the composites, but also provides space for adjusting electrical conductivity, permittivity, permeability or thickness to obtain desired EMI shielding performance.

Ferrites are ferromagnetic materials featuring high permeability and moderate resistivity. These characteristics are useful in variety of electronic devices, which take advantages at higher frequencies and heat resistance, lower price or greater corrosion resistance. Ferrites are composed of iron oxide and one or more other metals in chemical combination. Due to their dielectric low losses, low eddy currents and high magnetization, they are extensively used as RF transformers, inductors or in the cores of power supplies [14,15]. 

Carbon black (CB) is frequently used low cost, reinforcing filler mainly in rubber industry. In addition to carbon atoms, CB also contain low amount of chemically bonded hydrogen and oxygen functional groups. The primary spherical-like carbon black particles are connected together to generate three-dimensional chain-like structural aggregates [16]. Carbon black is applied in variety of articles such as tires, footwear, conveyor belts or mechanical goods, etc. 

Carbon nanotubes (CNT), the sp^2^ allotropes of chemically bonded carbon atoms, have attracted significant research interest ever since their discovery due to their outstanding properties. The exceptional chemical, physical and electrical characteristics of CNT offer exciting possibilities for nano-scale composites and high-tech applications. Carbon nanotubes can be defined as stack of graphene sheets rolled up forming concentric tube cylinders. Nanotubes generally have a high aspect ratio (ratio of length to diameter), so they can be considered as nearly one-dimensional structures [17]. 

Carbon fillers exhibit unique characteristics as low density, high permittivity, outstanding conductivity, good mechanical and thermal stability as well as excellent physical properties. These attributes make them suitable candidates for EMI shielding applications. Moreover, due their diverse structures, they exhibit higher or lower reinforcing character and improve mechanical and dynamic properties of polymer composites. These materials thus offer a great opportunity to manufacture a lot of varieties of polymer materials with tunable electrical, magnetic, optical and physical-mechanical properties.

The preparation and investigation of polymer composite shields by the application of ferrites and carbon-based fillers into various polymer matrices has been the interest of many experimental studies [18,19,20,21,22,23,24,25]. The results have demonstrated that composites filled with those fillers can effectively shield EMI. Although, many of them have been focused on testing of shielding effects at high frequencies, mostly within the X-band ranges (8.2–12.4 GHz) or even higher electromagnetic frequencies. Commonly used electronic equipment, as TV sets, radios or laptops, etc. emit electromagnetic waves at frequencies lower than 3 GHz. Thus, the investigation of EMI shielding effectiveness at low frequencies becomes also of high importance. 

In the study, manganese-zinc ferrite was first solely applied into acrylonitrile-butadiene rubber (NBR) based matrix. Then, magnetic filler was combined with carbon nanotubes and carbon black, respectively in certain ratios and EMI absorption shielding effectiveness of rubber composites was examined within low frequencies. The purpose of combination of ferrite and carbon fillers was mainly to improve mechanical properties of composites, as ferrite acts as inactive filler and usually deteriorates tensile characteristics, as tensile strength, tear strength or moduli. There was also interest in how a combination of ferrite with carbon fillers can affect absorption shielding effectiveness of composites as both carbon black and carbon nanotubes belong to the family of electrically conductive fillers. NBR is a specialty polar type rubber with good flexibility and elasticity and excellent oil resistance. When filled with carbon fillers, applicable physical-mechanical properties can be easily achieved. Easy processing in line with good tensile characteristics and design flexibility of the rubber-based composites can provide benefits over traditional materials that are limited by intensive time and labor processes.

## 2. Experimental

### 2.1. Materials

Acrylonitrile-butadiene rubber having trade name SKN 3345 and 31–35% content of acrylonitrile was supplied from Sibur International, Moscow, Russia. NBR based rubber batch filled with multi-walled carbon nanotubes (type NC7000, content of carbon—90%, length of tubes—1.5 µm, diameter of tubes—9.5 nm, specific surface area—250–300 m^2^/g) was provided from Nanocyl SA, Sambreville, Belgium. NBR based rubber batch filled with carbon black (type Vulcan XC72, specialty carbon black with moderate electrical conductivity, low sulfur and impurity level) was compounded in Vipo, a.s. Partizánske, Slovakia. Magnetic soft manganese-zinc ferrite used as magnetic filler was delivered from Epcos s.r.o., Šumperk, Czech Republic. The particle size and structural characteristics of the applied ferrite are summarized in Table 1. The distribution of particle size was determined using a better sizer ST instrument by authors. A standard sulfur-based curing system consisting of activators zinc oxide and stearic acid (Slovlak, Košeca, Slovakia), accelerator *N*-cyclohexyl-2-benzothiazole sulfenamide (Duslo, Šaľa, Slovakia) and sulfur (Siarkopol, Tarnobrzeg, Poland) was used for cross-linking of composites.

### 2.2. Methods

#### 2.2.1. Fabrication and Curing of Rubber Compounds

In the current work, three types of rubber formulations were prepared and tested. The amount of curing additives was kept at a constant level in all rubber formulations and only the type and content of the filler or fillers combination was changed. Manganese-zinc ferrite was first applied in composites in concentration scale ranging from 100 to 500 phr. In the second types of composites, the content of carbon black was kept constant in all composites—20 phr, and the amount of ferrite varied from 100 to 500 phr. The usual amount of carbon black in generally used rubber products ranges from 10 to 30 phr, thus, the amount 20 phr of CB was chosen as the basis for composite fabrication. Similarly, in the third composite types, carbon nanotubes at a constant level—5 phr were compounded with magnetic filler in concentration range 100–500 phr. In fact, lower amounts of carbon nanotubes are added into rubber formulations in order to achieve applicable functional properties (usually from 2 to 15 phr), and thus, the concentration of CNT was set up to 5 phr.

A laboratory kneading machine Brabender (Brabender GmbH & Co. KG, Duisburg, Germany) was used to fabricate rubber compounds. The temperature of compounding was set up to 90 °C with rotor speed 50 rpm. The compounding procedure lasted 13 min. During the preparation of rubber compounds filled only with ferrite, NBR was first plasticated for 2.5 min, then zinc oxide and stearic acid were added. After the next 2 min, filler was introduced. The first step mixing took 9 min overall. In the second step, which took for 4 min at 90 °C and 50 rpm, the accelerator and sulfur were applied. Finally, the rubber compounds were homogenized and sheeted in two-roll mill. 

The preparation procedure of rubber compounds filled with combination of ferrite and carbon-based fillers proceeded under similar conditions as in the previous case, but NBR/CB batch and NBR/CNT batch were first compounded with virgin NBR to reduce the amount of carbon black to 20 phr or carbon nanotubes to 5 phr. Then, the compounding steps were identical. 

The curing process of rubber compounds was performed at 160 °C under a pressure of 15 MPa using a hydraulic press—Fontijne (Fontijne, Vlaardingen, Holland). Finally, thin rubber sheets (thickness 2 mm, width 15 × 15 cm) of cured composites were achieved. 

#### 2.2.2. Investigation of Shielding Characteristics

The frequency dependencies of complex (relative) permeability *µ* = *µ′* − *jµ″* for toroidal samples were measured using combined impedance/network analysis method by means of a vector analyser (Agilent E5071C, Keysight Technologies, Santa Rosa, California, USA) in the frequency range of 1 MHz–3 GHz. During measurements, a toroidal sample was inserted into a magnetic holder (Agilent 16454A, Keysight Technologies, Santa Rosa, California, USA) and the complex permeability was evaluated from measured impedances *μ* = *μ′* − *jμ″* = 1 + (*Z* − *Z_air_*)/(*jhμ*_0_ *f* ln(*b/c*)), where *Z* and *Z_air_* are the input complex impedances of the 16454A holder with and without a toroidal sample, respectively, h is the height of the sample, *μ*_0_ = 4π⋅10^−7^ H/m is the permeability of free space, *f* is the frequency, and *b* and *c* are the outer and inner diameters of the sample.

The frequency dependencies of complex (relative) permittivity *ε* = *ε′* − *jε″* for disc samples were measured using a vector analyser Agilent E5071C, equipped with a dielectric holder (Agilent 16453A). The complex permittivity was computed from measured admittance *ε* = *ε′* − *jε″* = (*Y* × *h*)/(*jωε_o_S*), where *Y* is the input complex admittance of the 16453A holder with a disc sample, *h* is the height of the sample, *ε*_0_ = 8.854·10^−12^ F/m is the permittivity of free space, and *S* is the area of lower electrode. The electrical *dc* conductivity of composite materials was evaluated using standard two-probe method.

A high frequency single-layer electromagnetic wave absorption properties (return loss *RL*, matching thickness *d_m_*, matching frequency *f_m_*, bandwidth ∆*f* for *RL* at −10 dB and *RL* at −20 dB, and the minimum of return loss *RL_min_*) of composite materials were obtained by calculations of return loss *RL* = 20 log |(*Z_in_* − 1)/(*Z_in_* + 1)|, where *Z_in_* = (*μ*/*ε*)^1/2^tanh[(*jω·d/c*)(*μ*·*ε*)] is the normalized value of input complex impedance of the absorber, *d* is the thickness of the single-layer absorber (covered with a metal sheet), *c* is the velocity of light in vacuum. The composite absorbs maximum of the electromagnetic plane wave energy when normalized value of impedance *Z_in_* ≈ 1. The maximum absorption is then reached at a matching frequency *f* = *f_m_*, matching thickness *d* = *d_m_* and minimum return loss *RL_min_*.

#### 2.2.3. Evaluation of Thermal Characteristics of Composites

The Isomet appliance (Applied Precision Ltd., Bratislava, Slovakia) was in service in order to determine thermal parameters of composites. The measurement is based on the analysis of time dependence of thermal response to the impulses of the thermal flow into the analyzed material. The thermal flow is generated by scattered electric discharge in probe resistor, which is thermal-conductively connected with the analyzed material. The temperature of the resistor is scanned by the semiconductive detector. 

#### 2.2.4. Investigation of Mechanical Characteristics

The tensile characteristics of composites were measured by application of Zwick Roell/Z 2.5 equipment (Zwick Roell Group, Ulm, Germany) at a crosshead speed of 500 mm/min and room temperature in accordance with the valid technical standards. Dumbbell-shaped test specimens having thickness 2 mm, length 80 mm and width 6.4 mm were used for experiments.

#### 2.2.5. Microscopic Analysis

The microstructure and surface morphology of composites were observed using Jeol JSM-7500F (Jeol Ltd., Tokyo, Japan) scanning electron microscope (SEM). The samples were fractured into small fragments after cooling down in liquid nitrogen under glass transition temperature. The source of electrons is cold cathode UHV field emission gun, the accelerate voltage ranges from 0.1 kV to 30 kV and the resolution is 1.0 nm at 15 kV and 1.4 nm at 1 kV. SEM images are captured by a CCD-Camera EDS (Oxford INCA X-ACT).

## 3. Results and Discussion

### 3.1. Electromagnetic Shielding Characteristics of Composites Filled with Ferrite

The first part of the study was aimed at the preparation of composites filled with magnetic soft manganese-zinc ferrite into matrix based on NBR. Ferrite was dosed to the rubber compounds in concentration range 100–500 phr and the fabricated rubber compounds were subsequently cured at 160 °C by application of sulfur curing system. The optimum cure time t_c90_ of the reference unfilled sample was 8.5 min. The application of 100 phr ferrite into rubber formulation caused the reduction of the optimum cure time to 6 min. By next increasing content of ferrite, the t_c90_ slightly decreased to 4.5 min for the maximally filled composite. Electromagnetic characteristics and absorption shielding effectiveness of composites were investigated through determination of complex permittivity, complex permeability and return loss in frequency range from 1 MHz–3 GHz. As already outlined, generally used electronic equipment emit electromagnetic radiation within low frequency ranges, usually below 3 GHz. Therefore, investigation of electromagnetic and shielding characteristics within this frequency range is of strong interest.

**Figure 1:**
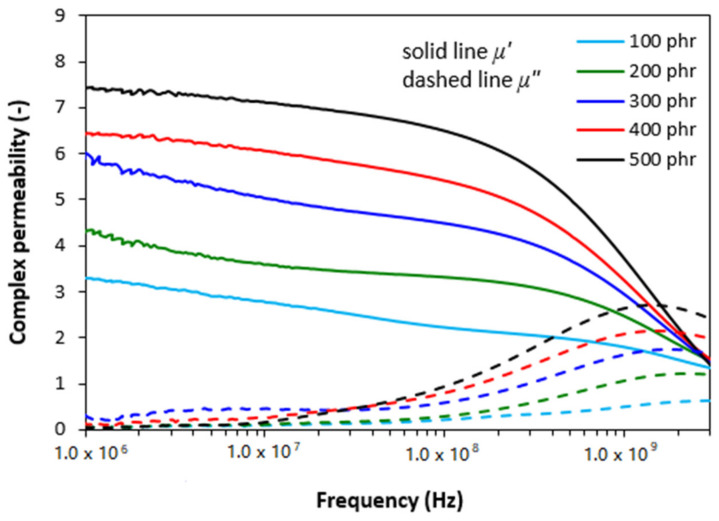
Frequency dependences of real *ε′* and imaginary *ε″* parts of complex permeability for ferrite filled composites.

The frequency dependencies of real and imaginary parts of complex permeability (*µ* = *µ′* − *jµ″*) for rubber magnetic composites are presented in Figure 1. As shown, there is only small change of the real part *µ′* with the change in frequency up to about 200 MHz, then it decreases close to one. The imaginary permeability *µ″* can be neglected up to about 100 MHz, then increases and after reaching a maximum at a resonance frequency *f_r_*, it drops down to a low value. The maximum in frequency dependences of imaginary permeability corresponds to the maximal permeability loss, i.e., magnetic loss. It also becomes apparent from Figure 1 that the real permeability of composites at low frequencies increased with increasing content of magnetic soft filler, from 3.3 for the composite filled 100 phr of ferrite to 7.5 for the maximally filled composite at 1 MHz. Then, the differences in *µ′* in dependence on ferrite content became smaller over 1 GHz. On the other hand, the differences in imaginary permeability of ferrite filled composites were not significant at low frequencies. Then, the values of *µ″* increased proportionally to the content of magnetic filler. Simultaneously, the resonance frequency *f_r_* decreased with ferrite content increasing, from about 3000 MHz for the composite filled with 100 phr of ferrite to around 1356 MHz for the composite with maximum ferrite content.

**Figure 2:**
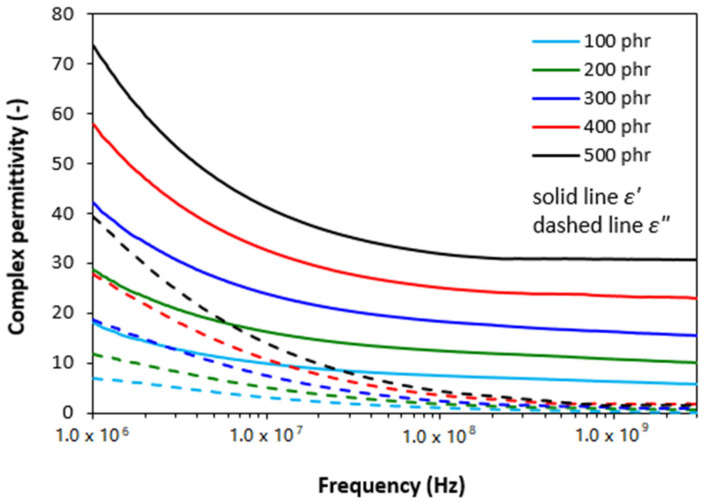
Frequency dependences of real *ε′* and imaginary *ε″* parts of complex permittivity for ferrite filled composites.

From frequency responses of complex permittivity of ferrite filled composites (Figure 2) it becomes obvious that the real part *ε′* first decreased with increase in frequency up to roughly 10 MHz. Then, it stabilized at constant values. The initial decrease of *ε′* can be attributed to the semiconducting nature of magnetic soft manganese-zinc ferrite. As also seen in Figure 2, the higher was the amount of magnetic filler in composites, the higher was the real permittivity. When the content of ferrite in composites increased from 100 to 500 phr, the real part increased from 18 to 74 at 1 MHz. With increasing frequency of EMI, the differences in real permittivity became less visible. The imaginary permittivity *ε″* of composites showed the similar frequency dependences. Upon initial decline at low frequencies, the differences in *ε″* were negligible over 1 GHz. The obtained changes in frequency dependencies of complex permittivity can be attributed to various polarization mechanisms, which are generated in ferrite as well as rubber matrix due to their dielectric nature (mostly polarization caused by space charges that accumulate at phase interfaces between the filler and the rubber matrix).

As already outlined, the shielding of EMI by absorption seems to be more desirable than refection shielding as the electromagnetic radiation is efficiently absorb by the shield and not accumulated in the surrounding. The absorbed electromagnetic radiation is then usually transferred in other forms of energy, as for instance to heat. The absorption shielding effectiveness of composites was examined by determination of return loss *RL* in decibels. Return loss provided information, in terms of which amount of incident EMI can be absorbed by the shielding material. It has been reported in scientic works that materials showing return loss at −10 dB can absorb 90–95% of EMI. The materials reaching return loss at −20 dB can efficiently absorb around 99% of electromagnetic radiation [26,27,28]. 

**Figure 3:**
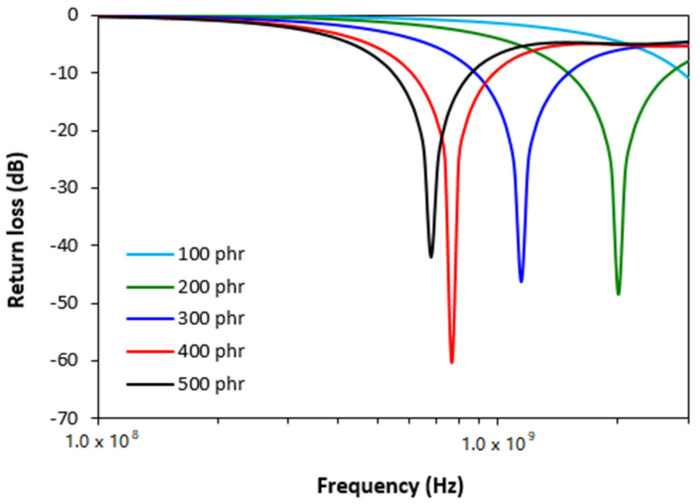
Frequency dependences of return loss for ferrite filled composites.

Figure 3 depicts the return loss of ferrite filled composites in the examined frequency range. It becomes apparent that all composites containing 200–500 phr of manganese-zinc ferrite exhibit suitable absorption shielding performance. As also shown, with increasing content of ferrite, the absorption shielding effectiveness and absorption maxima of composites shift to lower frequencies of EMI. As the best absorption composite shield can be considered the material filled with 200 phr of magnetic filler as it demonstrated return loss at −10 and −20 dB in the widest frequency range, i.e., from 1.5 to 2.7 GHz at −10 dB and from 1.85 GHz to 2.2 GHz at −20 dB. The absorption maximum of this shield was found to be at −48 dB at the frequency 2 GHz of the incident EMI. The composite containing 400 phr of ferrite filler showed lower absorption maximum (−60 dB), but also a lower frequency range with effective absorption of EMI (*RL* at −10 dB within 0.6–0.98 GHz frequency range and *RL* at −20 dB within only 0.72–0.83 GHz frequency range). The maximally filled composite demonstrated the lowest effective absorption bandwidth and the highest absorption maximum. The electromagnetic absorption characteristics (bandwidth Δ*f* for *RL* at −10 and at −20 dB, minimum value of return loss *RL_min_* at a matching frequency *f_m_* and matching frequency *f_m_*) are summarized in Table 2. It can be stated based upon the obtained results that ferrite filled rubber composites can be used as efficient EMI shields at frequencies above 0.5 GHz. 

### 3.2. Electromagnetic Shielding Characteristics of Composites Filled with Ferrite and Carbon Black

In the second part of the study, manganese-zinc ferrite was combined with carbon black in order to prepare EMI composite shields. The content of CB was kept constant in all composites—20 phr, while ferrite was incorporated into the rubber formulations in the amount ranging from 100 to 500 phr. The composites were cured at 160 °C and the increasing content of ferrite resulted to the decrease of optimum cure time from 15 min for the composite filled only with carbon black to 7 min for the composite filled with CB and 500 phr of ferrite. Complex permeability and complex permittivity as well as return loss of composites were first investigated in tested frequency range 1 MHz–3 GHz. 

**Figure 4:**
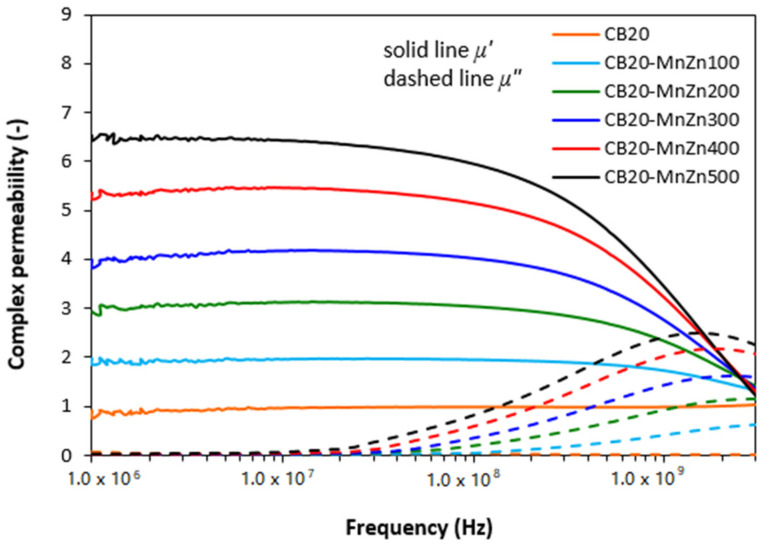
Frequency dependences of real *ε′* and imaginary *ε″* parts of complex permeability for hybrid CB/ferrite filled composites.

From graphical illustration of frequency dependences of complex permeability for composites filled with combination of ferrite and carbon black (Figure 4) it is possible to observe the similar frequency responses of both, real *µ′* and imaginary *µ″* parts as in the case of composites filled only with magnetic filler. As seen in Figure 4, the lowest real and imaginary permeability was found to have the composite filled only with carbon black and their values seem also to be independent on frequency. The increasing amount of magnetic filler in hybrid composites resulted in the increase of real permeability, mainly at lower frequencies and imaginary permeability at higher frequencies. Looking at Figure 1 and Figure 4, one can see that both, real and imaginary parts for hybrid CB/ferrite composites are slightly lower in comparison with equivalent composites filled only with magnetic filler, mainly at low frequencies. 

**Figure 5:**
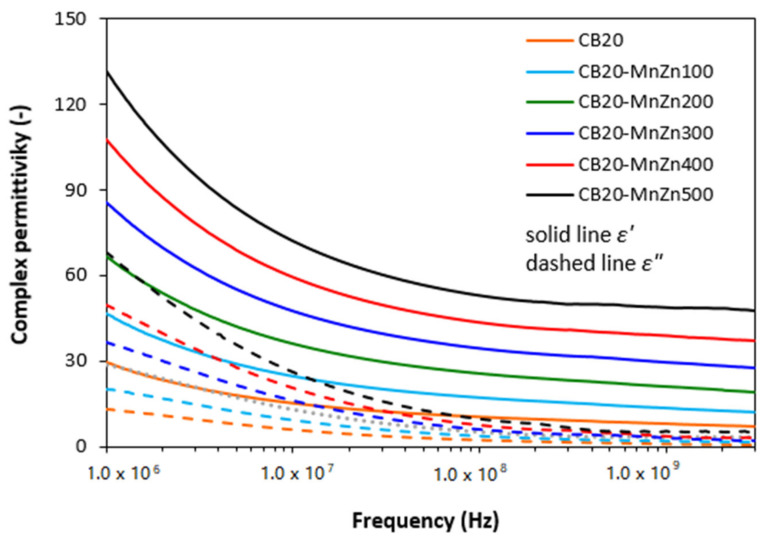
Frequency dependences of real *ε′* and imaginary *ε″* parts of complex permittivity for hybrid CB/ferrite filled composites.

As shown in Figure 5, the frequency dependences of real *ε′* and imaginary *ε″* permittivity for hybrid composites were also very similar as in the case of corresponding ferrite filled composites (Figure 2). It can also be stated that the lowest real and imaginary permittivity was found to have the composite filled only with carbon black. The increasing content of ferrite in hybrid composites resulted in the increase of both parts. The real permittivity first sharply decreased at frequencies up to about 10 MHz, then it settled on constant values. The *ε′* of the composite containing 100 phr of ferrite decreased from almost 47 to about 12 when the frequency changed from 1 MHz to 3 GHz. The increase in ferrite loading up to its maximum content resulted in the increase of real part up to nearly 132 at 1 MHz. Then, it decreased to 48 at 3 GHz. The similar trend was also recorded in frequency dependencies of imaginary part. The real permittivity of composites was higher in comparison with imaginary part in the whole tested frequency range. The value of *ε″* for the maximally filled composite decreased from 68 at 1 MHz to 5 at maximum tested frequency. It also becomes apparent that the real and imaginary permittivity of hybrid composites provide higher values when compared to equivalent composites filled only with magnetic filler. 

**Figure 6:**
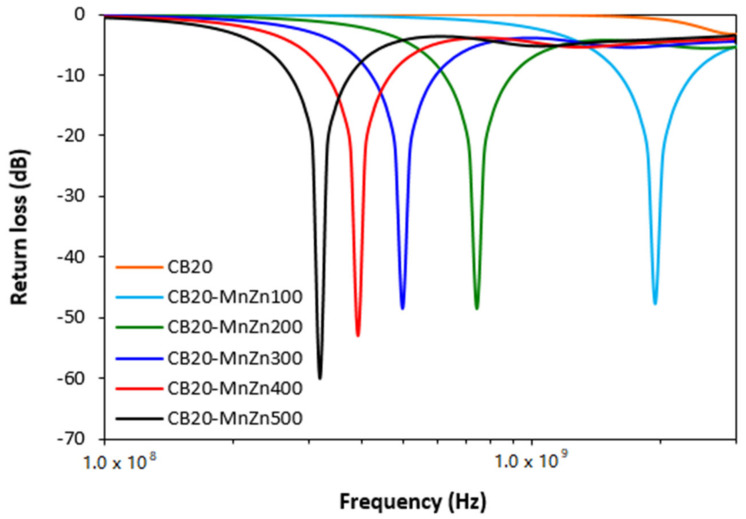
Frequency dependences of return loss for hybrid CB/ferrite filled composites.

From frequency dependences of return loss for hybrid CB/ferrite filled composites (Figure 6) it becomes apparent that the composite filled only with carbon black does not provide any absorption shielding effectiveness, because it did not reach the return loss at least at −10 dB. With exclusion of CB filled composite, all hybrid composites exhibited EMI absorption shielding performance. As the best absorption shield can be considered the composite filled with 100 phr of ferrite, as this shield exhibited return loss at −10 dB in the widest frequency bandwidth, i.e., from 1.6 to 2.35 GHz. The absorption maximum was detected at −48 dB at frequency 1.9 GHz of electromagnetic radiation. The lowest return loss demonstrated the maximally filled composite (−60 dB). However, this composite also showed the lowest efficient absorption frequency range at −10 and −20 dB, as also seen in Table 3. It becomes obvious from Figure 6 and Table 3 that increasing content of ferrite resulted in lower absorption maxima and the total absorption shielding effectiveness of hybrid composites moved to lower frequencies. In addition, with the increase in ferrite content, the effective absorption bandwidth of composites became narrower. The calculated values of absorption parameters, presented in Table 3, indicate that hybrid CB/ferrite composites can be applied in EMI shielding applications at frequencies over 0.3 GHz. When comparing composites filled only with magnetic filler (Figure 3, Table 2) and hybrid CB/ferrite composites (Figure 6, Table 3) it can be observed that composites filled with combination of ferrite and carbon black show lower absorption maxima, but also lower matching frequencies and narrower frequency ranges for *RL* at −10 dB and −20 dB. It can be stated that the combination of ferrite and carbon black caused the shifting of effective absorption shielding ability to lower frequencies of EMI. On the other side, narrower absorption peaks of hybrid composites point out to the fact that absorption shielding performance of those composites is lower in comparison with equivalent ferrite filled composites.

### 3.3. Electromagnetic Shielding Characteristics and Electrical Conducitity of Composites Filled with Ferrite and Carbon Nanotubes

Following study was focused on the fabrication and investigation of EMI shielding characteristics of composites filled with combination of ferrite and carbon nanotubes. The content of CNT was kept on constant level −5 phr, while magnetic filler was again incorporated into rubber matrix in the amount ranging from 100 to 500 phr. The optimum cure time of CNT/ferrite composites fluctuated in the range 13 to 18.5 min almost independently on the ferrite content.

**Figure 7:**
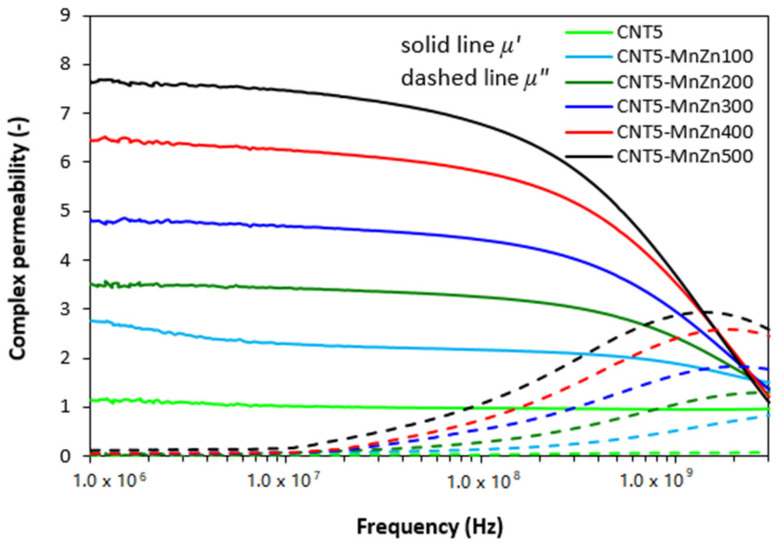
Frequency dependences of real *ε′* and imaginary *ε″* parts of complex permeability for hybrid CNT/ferrite filled composites.

The results obtained from determination of complex permeability (Figure 7) revealed that the lowest values of real and imaginary permeability exhibited the composite filled only with CNT, with almost no dependence of both parts on radiation frequency. The increasing degree of ferrite loading resulted in the increase of real permeability mainly at low frequencies and imaginary part at higher frequencies. When comparing the frequency dependences of complex permeability for composites filled only with magnetic filler (Figure 1) and hybrid composites filled with ferrite and carbon-based fillers (Figure 4 and Figure 7), it becomes apparent that the differences are very small and generally it can be stated that no significant changes in complex permeability of composites can be observed in dependence on the filler’s composition. 

**Figure 8:**
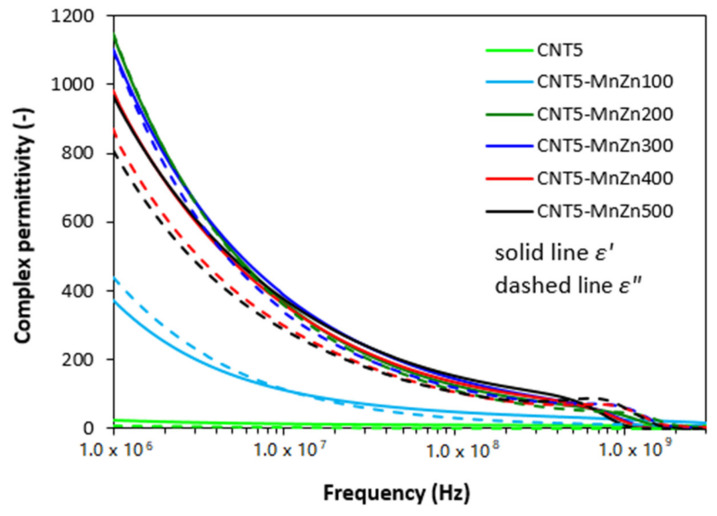
Frequency dependences of real *ε′* and imaginary *ε″* parts of complex permittivity for hybrid CNT/ferrite filled composites.

As shown in Figure 8, the real *ε′* and imaginary *ε″* parts of complex permittivity for hybrid CNT/ferrite composites showed significant decreasing trend with increase in radiation frequency. In comparison with the composite filled only with 5 phr of CNT, the application of 100 phr ferrite resulted in the increase of *ε′* from 23 up to 371 at 1 MHz. The increasing loading of ferrite in hybrid composites led to the increase of real permittivity up to 200 phr at 1 MHz (*ε′* = 1147 for the composite filled with 200 phr of ferrite and 5 phr of CNT). Subsequently, the real permittivity slightly decreased with next increasing content of magnetic filler at 1 MHz (*ε′* = 966 for the composite filled 500 phr of ferrite and 5 phr of CNT). Then, the increase in frequency from 1 MHz to 3 GHz caused the decrease of the real permittivity to very low values. As seen, the real permittivity of composites decreased to 17 or 0.1, respectively, by increasing content of magnetic filler from 100 phr up to maximum content at 3 GHz. The similar tendency can be also observed in frequency dependencies of imaginary part. It becomes also obvious from Figure 8 that the values of imaginary permittivity are very similar with real part *ε′* of equivalent composites in the whole tested frequency range. The incorporation of 100 phr magnetic filler resulted in the increase of imaginary permittivity in more than 430 at 1 MHz (from about 9 for the composite filled with 5 phr of CNT up to 439 for the composite filled with 5 phr of CNT and 100 phr of magnetic filler). The imaginary permittivity of the composite filled with 100 phr of ferrite dropped down to roughly 7 by increasing of frequency up to 3 GHz. The imaginary part of the maximally filled composite declined from 807 at 1 MHz to 1.5 at 3 GHz. When comparing the complex permittivity of composites filled only with manganese-zinc ferrite (Figure 2), hybrid CB/ferrite filled composites (Figure 5) and hybrid CNT/ferrite filled composites (Figure 8) it becomes apparent that both, the real and imaginary permittivity increase in the following order: ferrite composites < CB/ferrite composites < CNT/ferrite composites.

**Figure 9:**
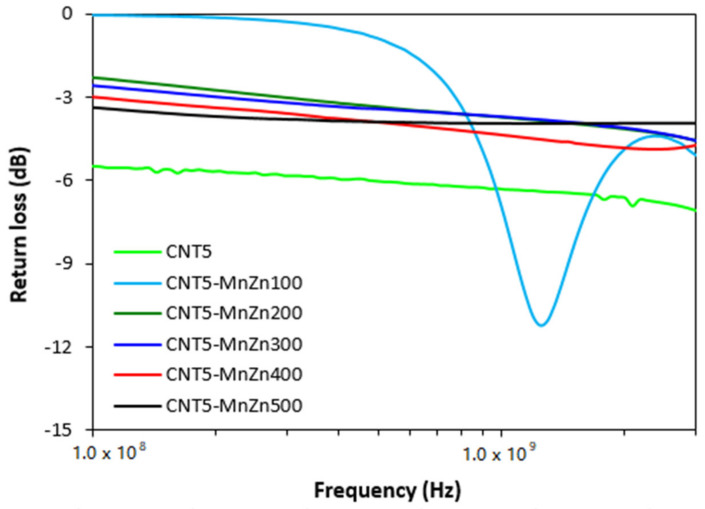
Frequency dependences of return loss for hybrid CNT/ferrite filled composites.

From graphical illustrations of frequency dependences of return loss for hybrid CNT/ferrite composites (Figure 9) it is shown that the composite filled only with carbon nanotubes and hybrid composites containing 5 phr of CNT and 200–500 phr of ferrite do not provide any absorption shielding performance, because they did not reach return loss at least at −10 dB. Only the composite filled with 5 phr of CNT and 100 phr of ferrite was found to have a slight absorption shielding ability. Although, the effective absorption frequency bandwidth of this composite at −10 dB was narrow and ranged only from 1.14 to 1.39 GHz. The absorption maximum was at −11.2 dB at frequency 1.24 GHz of EMI. 

The complex permeability and complex permittivity have been reported to be very important parameters, which influence the EMI shielding effectiveness. Parameter *µ′* represents magnetic storage capacity, whereas imaginary permeability *µ″* indicates magnetic dissipation or losses [29,30]. In generally, materials with high permeability have been shown to provide good shielding efficiency by absorption. Looking at Figure 4 and Figure 7, one can see that the composites filled only with carbon black or carbon nanotubes exhibit low real permeability and negligible imaginary permeability. In addition, the values of *µ′* and *µ″* seem to be independent on radiation frequency. Low values of complex permeability for CB and CNT filled composites are clearly reflected in non-magnetic character of carbon-based fillers. The incorporation of manganese-zinc ferrite possessing magnetic dipoles resulted in the increase of complex permeability of ferrite filled composites as well as both types of hybrid composites. Both parts of permeability were also found to be frequency dependent. The similar values of complex permeability of all types of tested composites clearly demonstrate that frequency dependences of real and imaginary permeability are dependent only on the content of ferrite, regardless of the type and amount of carbon-based fillers. 

The main mechanisms that determine the permeability consist of spin precession, domain wall movement, hysteresis loss and eddy current effect [28]. The hysteresis loss due to the irreversible magnetization can be neglected as the composite materials were tested in Rayleigh region (of low magnetic fields), and at higher frequencies. The spin precession and the domain wall movement are usually connected with resonance phenomena in the permeability spectrum, namely the domain wall and the spin precession (or natural ferromagnetic) resonance. The observed peaks in frequency dependencies of imaginary permeability correspond to the spin precession resonance only as the domain walls are unable to keep pace with *ac* electromagnetic field over about 10^8^ Hz. Eddy current effect might also contribute to permeability loss due to high *dc* electrical conductivity and low particle size of fillers. 

The real part of complex permittivity *ε′* represents the electrical charge storage capacity in the material and can be understood as the amount of polarization centers and micro-capacitors [31]. It is mainly influenced by the polarization (formation of localized charges) within the composite system. Polarization of the conducting filler, rubber matrix as well as interfacial polarization can occur in dependence on frequency range [32,33]. Micro-capacitors are formed by particles or aggregates of the fillers that act as electrodes filled with insulating rubber matrix, while various defects in fillers structure provide space for polarization centers. It can be stated that the increase of micro-capacitors and structural defects with increasing content of ferrite is responsible for the increase in real permittivity of composites. Higher values of real permittivity for hybrid composites can be attributed to the presence of carbon-based fillers with higher conductivity and much higher charge storage capacity. Moreover, the increase in filler loading leads to the reduction of the gap between the filler particles. Even, the presence of small amount of carbon-based fillers can significantly reduce the dimension between the filler particles due to the structural aggregates of carbon black and cylindrical shape of carbon nanotubes with high aspect ratio. This leads to the increase of the polarization of the rubber matrix filling the gap between filler particles. Significant increase in real permittivity of hybrid composites filled with 5 phr of CNT and 100 phr ferrite (*ε′* = 371) when compared to the composite filled only with 5 phr of CNT (*ε′* = 23) or 100 phr of ferrite (*ε′* = 18) might be caused by reaching of percolation threshold by combination of both fillers (connecting of filler particles within the rubber matrix and formation of filler conductive paths). The outstanding increase of real permittivity at low frequencies is likely caused by micro-capacity and polarization of the fillers and filler-rubber interfacial charge polarization. 

Imaginary permittivity correlates with dissipation of electrical energy (dielectric loss). It is influenced by complex phenomena: ionic, electronic, dipole polarization, interfacial polarization, natural resonance and related relaxation phenomena [34,35]. As already outlined, interfacial and space charge relaxations occur because charge carriers are trapped at the interfaces of heterogeneous composite system [36]. In generally, the increasing amount of conductive filler results in the increase of the number of conductive networks within the composites, which is beneficial for higher imaginary permittivity and consequently for higher values of complex permittivity [37,38]. Conductive filler networks can also act as dissipating mobile charge carriers. The relation between imaginary permittivity and electrical conductivity can be expressed as:

ε″=σ_dc_/2πε_0_f

where *ε″* is imaginary part of permittivity, *ε_o_* is permittivity of the free space (*ε_o_* = 8.854 × 10^−12^ F/m), *σ_dc_* is electrical conductivity (S/m) and *f* is frequency (Hz). 

**Figure 10:**
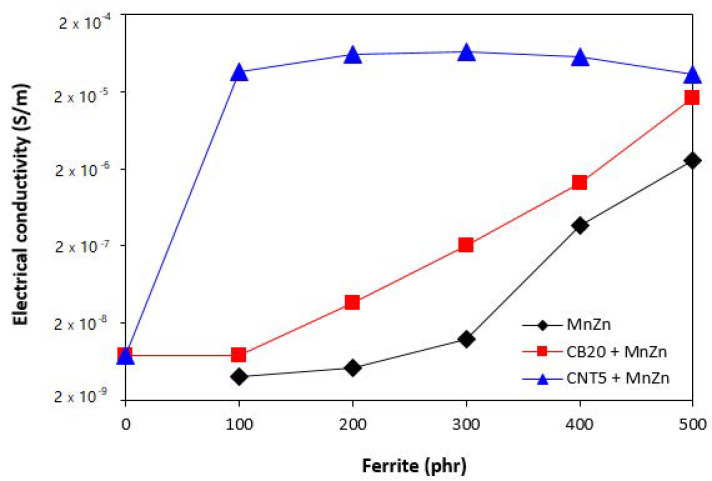
Electrical conductivity of composites.

The electrical conductivity of composites filled only with ferrite and hybrid composites is depicted in Figure 10. As shown, the lowest electrical conductivity exhibited composites filled only with manganese-zinc ferrite. It also becomes obvious that the increasing content of ferrite resulted in the increase of electrical conductivity of composites despite the fact that ferrite ranks among dielectric materials. Composites filled with combination of ferrite and carbon-based fillers exhibited higher electrical conductivity compared to equivalent composites filled only with ferrite. As also seen in Figure 10, the electrical conductivity of composites filled only with 20 phr of carbon black or 5 phr of carbon nanotubes was almost the same. The incorporation of 100 phr magnetic filler resulted in a significant increase of electrical conductivity of hybrid CNT/ferrite composites. Based upon the achieved results it might be stated that by combination of carbon nanotubes and magnetic filler, the percolation threshold and formation of conductive filler paths are reached even at low magnetic filler content resulting in outstanding increase of electrical conductivity. When comparing graphical illustrations of permittivity of composites filled only with ferrite (Figure 2), hybrid CB/ferrite composites (Figure 5) and CNT/ferrite composites (Figure 8) and their electrical conductivity, one can see, that the higher is electrical conductivity, the higher is the imaginary permittivity and consequently, the higher is complex permittivity of composites, mainly at low frequencies. On the other hand, looking at the mutual relation between imaginary permittivity and radiation frequency, it becomes apparent that the increasing frequency of electromagnetic radiation causes the decrease in imaginary permittivity, as was also confirmed during experimental measurements. 

The experimentally obtained results also revealed that absorption shielding efficiency of composites decreased in the following order: ferrite composites < CB/ferrite composites < CNT/ferrite composites. The reason can be attributed to the enhanced electrical conductivity and complex permittivity of composites, which increased in the same order. It has been reported that materials with high electrical conductivity are suitable candidates for EMI shielding applications based on reflection, mainly at low frequencies [39,40,41,42].

### 3.4. Thermal Properties, Mechanical Characteristics and Morphology of Composites

**Figure 11:**
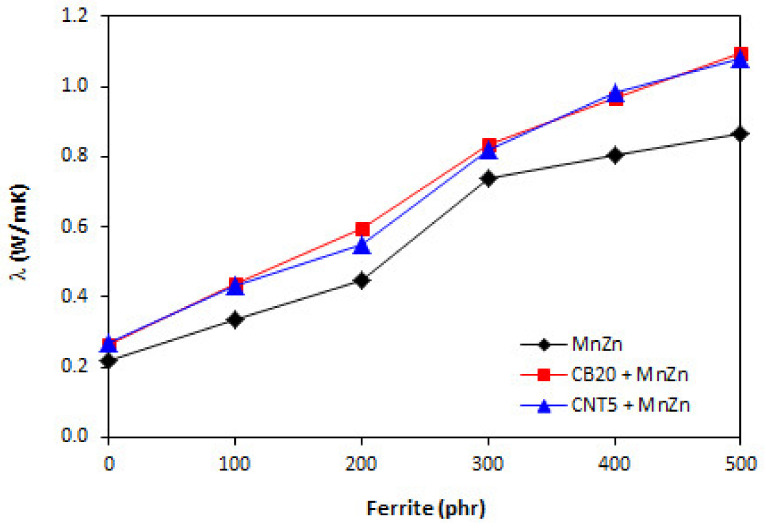
Thermal conductivity coefficient *λ* of composites.

**Figure 12:**
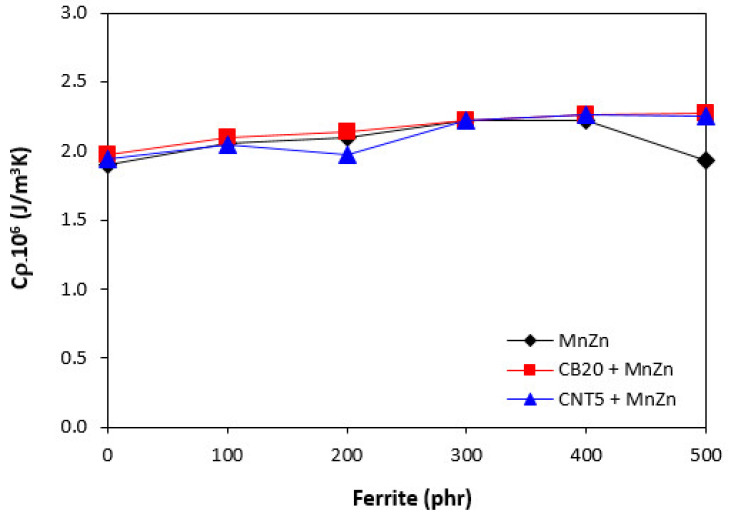
Volumetric heat capacity *Cρ* of composites.

Polymers in generally have low thermal conductivity. As the thermal conductivity of ferrites and carbon-based fillers is much higher, their incorporation into rubber matrix should lead to the increased thermal flow through composite systems. Thermal conductivity refers to heat built up and heat transfer trough the material. The higher thermal flow can result to faster heating of rubber compounds, consequently to faster vulcanization. On the other hand, during mechanical and dynamic strain and accumulation of energy, the generated heat can be more easily lead away from the material. The results obtained from determination of thermal conductivity coefficient *λ* confirmed the presumption as shown in Figure 11, seeing that the higher was the amount of magnetic filler, the higher was the thermal flow through the composites. It can also be stated that the thermal conductivity coefficient of the composites filled only with 20 phr of CB or 5 phr of CNT was almost the same. The incorporation of ferrite resulted in further increase of coefficient *λ* with the similar values for both types of hybrid composites. By contrast, the volumetric heat capacity *Cρ* of composites was almost independent on the content of ferrite or combination of fillers and its values fluctuated only in the low experimental range (Figure 12).

**Figure 13:**
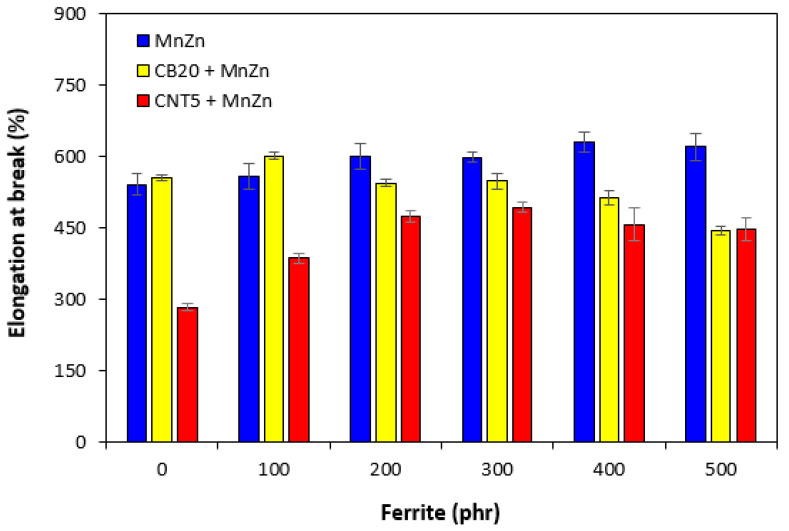
Elongation at break of composites.

**Figure 14:**
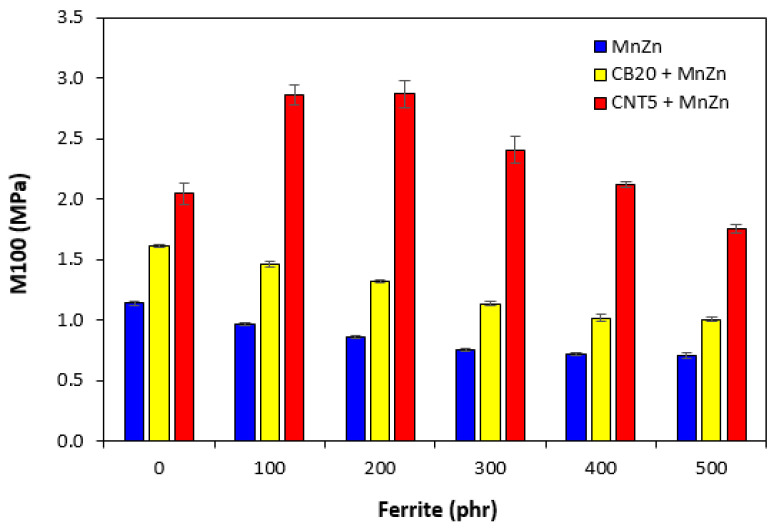
Modulus M100 of composites.

**Figure 15:**
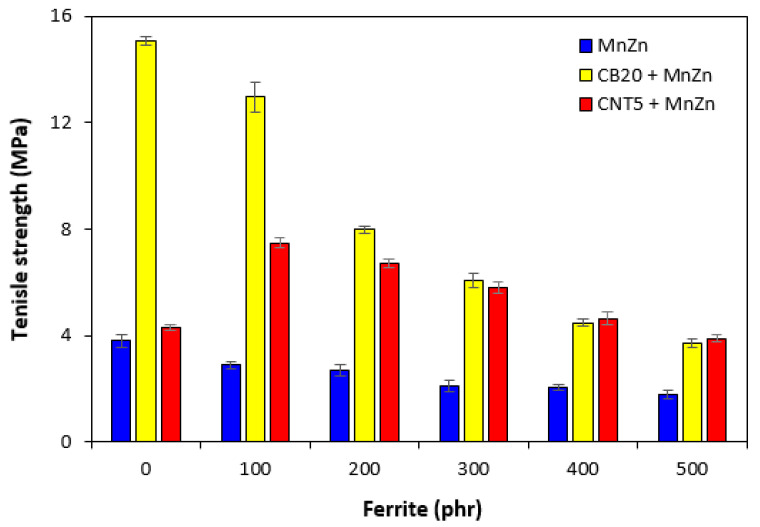
Tensile strength of composites.

The mechanical properties of composites filled only with ferrite and hybrid composites are graphically illustrated in Figure 13, Figure 14 and Figure 15. As shown in Figure 13, the elongation at break of ferrite filled composites showed slight increasing tendency with increasing content of magnetic filler. On the other hand, upon initial increase of the property of hybrid CB/ferrite composites at 100 phr of ferrite, the elongation at break of those composites showed decreasing trend with next increase in ferrite content. The lowest elongation at break exhibited hybrid CNT/ferrite composites. It is also possible to observe that elongation at break composites filled with combination of ferrite and carbon nanotubes passed over a maximum at 300 phr of ferrite. Then, a decreasing trend was observed. The elongation at break of the maximally filled CNT/ferrite composite was still more than 150% higher when compared to the composite filled only with 5 phr of CNT. It also becomes very interesting that while the elongation at break of the composite filled with 20 phr of CB was almost two times higher in comparison with that of the composite filled with 5 phr of CNT, the elongation at break of the maximally filled CNT/ferrite and CB/ferrite composites was established almost on the same value. From Figure 14, it becomes apparent that the lowest modulus M100 were found to have composites filled only with ferrite. The increasing amount of magnetic filler in composites resulted in a slight decrease of M100. The increasing content of ferrite caused also the decrease in M100 of hybrid CB/ferrite composites, although the modulus of hybrid CB/ferrite composites was higher when compared to the equivalent ferrite filled composites. The highest M100 were found to have hybrid CNT/ferrite composites. When compared to the composite filled only with 5 phr of CNT, the incorporation of 100 and 200 phr ferrite resulted in the increase in M100 of CNT/ferrite composites. Then, the modulus showed decreasing tendency with next increasing content of ferrite. Similarly, the lowest tensile strength exhibited composites filled only with magnetic filler (Figure 15). In addition, the higher the amount of magnetic filler in composites, the lower was the tensile strength. The application of CB and CNT resulted in the increase of tensile strength. The biggest difference in tensile strength among all types of composites was recorded for the reference samples and composites with lower ferrite content. As seen in Figure 15, the highest tensile strength demonstrated the composite filled with 20 phr of CB and the composite filled with combination of carbon black and 100 phr of ferrite. The tensile strength of the hybrid CNT/ferrite composite with 100 phr of ferrite first increased in almost 3 MPa when compared to the reference CNT filled composite (from 4.3 MPa for the composite filled with 5 phr of CNT to nearly 7.5 MPa for the composite filled with carbon nanotubes and 100 phr of ferrite). Then, the tensile strength of hybrid CNT/ferrite composites was found to decrease with next increasing content of magnetic filler. The initial enhancement of tensile strength for hybrid CNT/ferrite composites can be ascribed to some synergistic effect of both, carbon nanotubes and ferrite. It also becomes obvious that the higher the content of ferrite in all types of composites, the lower was the difference in tensile strength, but the tensile strength of composites filled with combination of carbon-based fillers and ferrite was higher in all ferrite concentration scale when compared to the corresponding composites filled only with manganese-zinc filler. 

**Figure 16:**
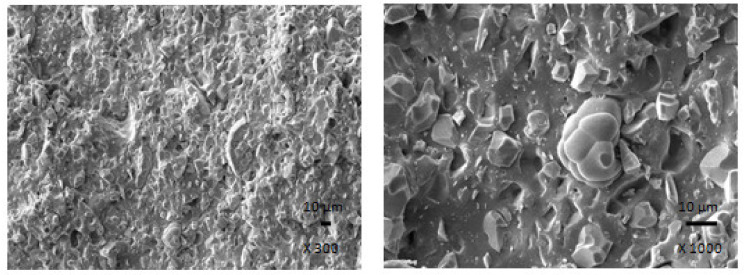
SEM images of composite containing 300 phr of ferrite.

**Figure 17:**
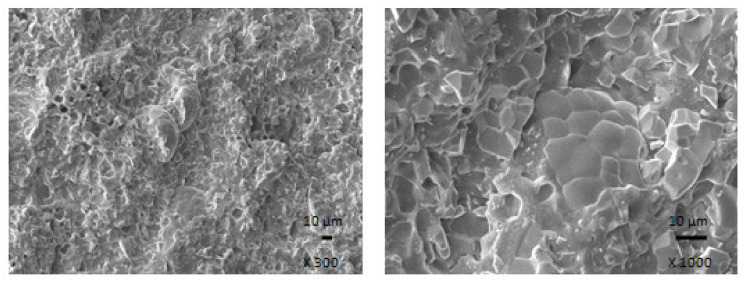
SEM images of hybrid CB/ferrite composite containing 300 phr of ferrite.

**Figure 18:**
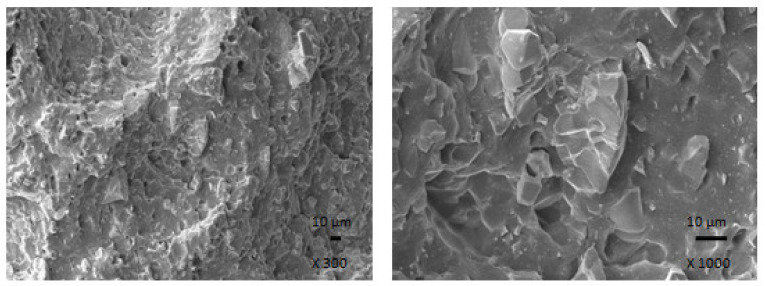
SEM images of hybrid CNT/ferrite composite containing 100 phr of ferrite.

The results clearly demonstrated that ferrite acts as inactive filler when incorporated in the rubber matrix. The reason can be attributed to the poor compatibility and adhesion between ferrite and rubber on the filler-rubber interface, as was also confirmed from SEM analysis of composites (Figure 16). In addition to various voids and inhomogeneities existing on the interface filler-rubber, it is also agglomeration of ferrite particles, which contributed to the worsening of adhesion between both components. On the other hand, the introduction of carbon-based fillers resulted in the improvement of mechanical characteristics of hybrid composites. Both fillers exhibit strong reinforcing effect when they are incorporated into rubber matrices. Moreover, the dispersion of fillers, mainly ferrite in hybrid CB/ferrite composites (Figure 17) as well as CNT/ferrite composites (Figure 18) was higher, which can be attributed to higher viscosity of the rubber compounds owing to the presence of carbon-based fillers. Thus, higher shear stress was generated during fabrication and compounding of composites, which facilitated distribution and dispersion of ferrite inside the rubber matrix. It also becomes apparent from SEM images that both carbon-based fillers contributed to the improved adhesion and mutual compatibility between ferrite and the rubber matrix. 

## 4. Conclusions

Manganese-zinc ferrite alone and in combination with carbon fillers (CB and CNT) was incorporated into NBR based rubber matrix in order to prepare composite EMI shields. The influence of fillers on mechanical, thermal and electrical properties of composites was investigated. Electromagnetic parameters and absorption shielding ability of composites were examined within low frequencies. 

The results demonstrated that permeability of ferrite and hybrid composites was dependent only on the content of magnetic filler, regardless of the type and amount of carbon fillers. The electrical conductivity and permittivity of composites increased in the order: ferrite composites < CB/ferrite composites < CNT/ferrite composites. On the other hand, in the same order, it decreased the absorption shielding ability of composites. Based upon the achieved results, clear correlation among electrical conductivity, permittivity and absorption shielding ability of composites has been established. This means, the higher the electrical conductivity, the higher is permittivity and the lower is the absorption shielding effectiveness of composites within the frequency range 1 MHz–3 GHz. It was also shown during experiments that ferrite acts as inactive filler and aggravates mechanical properties of composites. The application of carbon-based filler resulted in the reinforcement of rubber matrix, better dispersion and distribution of ferrite and subsequently to the improvement of mechanical properties of composites. The enhanced mechanical characteristics can contribute to the broadening of application potential field of composite EMI shields. As ferrites have been found to be good absorbers of EMI, but inactive fillers from the point of physical-mechanical properties, the combination of ferrites with carbon-based fillers seems to be the future trend of effective EMI shields fabrication. Elastomer based composite shields can be applied in practical applications where good elasticity and flexibility together with improved mechanical properties are required. 

## Figures and Tables

**Table 1:** Particle size and characteristics of ferrite.

Characteristics	Values
Particle size (µm)	0.1–30
Specific surface area (m^2^/g)	10.99
Total porosity (%)	59.72
Density (g/cm^3^)	4.87
Electrical resistivity (Ω·m)	3

**Table 2:** Electromagnetic absorption characteristics of ferrite filled composites.

Ferrite (phr)	*RL_min_* (dB)	*f_m_* (MHz)	Δ*f* (MHz) for RL at −10 dB	Δ*f* (MHz) for RL at −20 dB
100	—	—	—	—
200	−48	2010	1220	370
300	−46	1148	630	188
400	−60	769	380	115
500	−42	682	310	103

**Table 3:** Electromagnetic absorption characteristics of hybrid CB/ferrite filled composites.

Ferrite (phr)	*RL_min_* (dB)	*f_m_* (MHz)	Δ*f* (MHz) for RL at −10 dB	Δ*f* (MHz) for RL at −20 dB
100	−48	1931	750	158
200	−49	739	270	73
300	−49	495	170	54
400	−53	390	140	42
500	−60	319	110	21

## Data Availability

Not applicable.
